# Caloric Restriction Effect on Proinflammatory Cytokines, Growth Hormone, and Steroid Hormone Concentrations during Exercise in Judokas

**DOI:** 10.1155/2015/809492

**Published:** 2015-05-14

**Authors:** Salma Abedelmalek, Hamdi Chtourou, Nizar Souissi, Zouhair Tabka

**Affiliations:** ^1^Department of Physiology, Sousse Faculty of Medicine, Avenue Mohamed Karoui, 4002 Sousse, Tunisia; ^2^Research Laboratory “Sports Performance Optimization” National Center of Medicine and Science in Sports (CNMSS), Tunis, Tunisia

## Abstract

The aim of this study was to evaluate the effect of caloric restriction on the immune and hormonal responses during exercise in judo athletes. In a randomised order, 11 male judokas (age: 20.45 ± 0.51; height: 1.71 ± 0.3 m; and body weight: 75.9 ± 3.1 kg) participate in this study during a period of weight maintenance (baseline) and after 7 days of caloric restriction (CR). All subjects performed the Special Judo Fitness Test (SJFT) during the two conditions. Values for nutrient intakes were obtained from a 7 d food record kept during a period of weight maintenance and after a 7-day food restriction (−5~6 MJ/day). Our results showed that CR resulted in significant decreases in body weight (*P* < 0.05) and performance (*P* < 0.05). However, heart rate and SJFT index (*P* < 0.05) increase significantly during CR in comparison to baseline. Moreover, exercise leads to a significant increase in testosterone, cortisol, growth hormone (GH), leukocytes, neutrophils, TNF-*α*, and IL-6, in both CR and baseline conditions. Compared to baseline, TNF-*α* and IL-6 were significantly higher during CR condition (*P* < 0.05). Additionally, CR leads to an increase in cortisol and GH (*P* < 0.05) and a decrease in testosterone concentrations (*P* < 0.05).

## 1. Introduction

In judo competition, division by weight class guarantees matched strength, agility, and power between the competitors [1, 2]. To be successful in international competitions, judo athletes must maintain their daily body weight within the limits of their class before competition. However, few athletes maintain their daily body weight within the limits of their class before competition. In general, most athletes rely on the rapid weight reduction. This rapid weight loss typically starts 3–5 days before competition, when athletes may restrict food with combination of intensive exercise in rubber or plastic suits, vomit, and use of saunas or diet pills [2, 3]. However, these weight-loss procedures can lead to hormonal imbalance [4], hyperthermia [5], cardiovascular distress [6], and impaired immune function [7, 8]. Importantly, these physiological alterations can decrease anaerobic capacity, an important determinant of overall performance in judo [9, 10].

On the other hand, pervious reports showed that strenuous high-intensity exercise induces immune suppression and may explain the increased risk of infection in athletes [11, 12]. Likewise, Meckel et al. [12] showed that two types of sprint interval sessions (i.e., increasing (100, 200, 300, and 400 m) and decreasing (400, 300, 200, and 100 m) distance) lead to a significant increase in the circulating pro- and anti-inflammatory mediators (i.e., IL-1, IL-6, and IL1ra) as well as GH, IGF-I, and testosterone levels. More recently, Abedelmalek et al. [13] showed a significant increase of IL-6 immediately after 30 s of Wingate test.

It is also well established that restriction of energy and nutrients results in a compromised immune system and, hence, a decreased resistance to infection. Likewise, CR appears to synchronize the central pacemaker in the SCN, suggesting a role for a metabolic state imposed by low calories in central clock entrainment. There is a general perception among athletes, coaches, and team physicians that athletes are more vulnerable to infections disease. Moreover, rapid weight reduction by energy restriction may have additional disadvantages in athletes who are in a chronic immunosuppressive state. In this context, Shimizu et al. [14] showed that 2 weeks of weight loss before a competition can impair cell-mediated immune function and induce high susceptibility to upper respiratory tract infections (URTIs) in judo athletes.

Results of several studies have suggested adverse effects of weight loss, including intensive training and energy restriction, on immune parameters in athletes. Weight loss reportedly reduces neutrophil phagocytic activity [15], T-cell proliferation and cytokine production such as interferon-g [16], and leukocyte counts [8]. Some athletes undergoing weight loss are known to suffer from infections. Nevertheless, these alterations depend on the type of weight loss (i.e., gradual or rapid weight loss), of the sport practised (i.e., aerobic versus anaerobic), of the intensity of the exercise, of the type of diet (i.e., high fat or high carbohydrate diet), and of the amount lost. Moreover, many negative effects of weight loss are reported by the authors; it is important to note that these responses depend on the amount lost. However, the data are controversial [17, 18]. Numerous attempts have been made to investigate the effects of exercise training or energy restriction on neutrophil functions [7, 19]. Thus, judo is often considered to be an explosive sport which demands great anaerobic strength and capacity [20], accompanied by a well-developed aerobic system [21].

Additionally, studies investigating the effects of weight loss on performance utilized laboratory-based techniques, which may not reflect the demands of real judo combat. Therefore, a judo-specific performance test (Special Judo Fitness Test, SJFT), which is more representative of judo movements than laboratory tests, has been proposed as a valid and reliable measure of performance in judo athletes [22, 23]. However, no report has described a study of the influences of weight loss on proinflammatory cytokines and steroid hormones during intensive and specific exercises. Likewise, little information about judo demand and weight loss practices in judo athletes, especially concerning the test used to represent judo performance, was available.

It is crucial, therefore, to investigate the effect of 7 days' food restriction on proinflammatory cytokines and steroid hormones in male judo athletes preparing for a national championship. In view of the above consideration, the purpose of this study was to determine the effects of caloric restriction on IL-6, TNF-*α*, testosterone, and cortisol during SJFT in judo athletes. In light of the literature observations, we hypothesized that CR affects the proinflammatory cytokines and steroid hormone responses to the SJFT.

## 2. Materials and Methods

### 2.1. Participants

Eleven healthy judo athletes (mean (±SD); age: 20.45 ± 2.51 years; height: 171.6 ± 3.6 cm; and weight 75.9 ± 3.1 kg) participated in the study. After receiving a thorough explanation of the protocol, they gave written consent to participate in this study. This study protocol was in accordance with the Helsinki Declaration for human experimentation and was approved by the university ethics committee. The participants were also selected based on their chronotype and on the basis of their answers to Horne and Ostberg [24] self-assessment questionnaire. They had an intermediate chronotype (i.e., sleep duration between 22:30 ± 1:00 and 07:00 ± 1:00 h) and kept standard times for eating prior to the commencement of the study (breakfast at 07:00 ± 1:00 h, lunch at 12:00 ± 1:00 h, and dinner at 20:00 ± 1:00 h). Their mean period of practising judo was 11.2 ± 3.1 years. The participants' technical levels were second and third Dan black belts. They competed in categories between −73 and 80 kg weight category. Based on the results of a self-reported questionnaire, no subject had been treated with any drug that is known to affect immune function, had experienced acute illness from infection during the first three months, or had smoked tobacco regularly. Subjects reported no sleep disorder and did not consume caffeine or any alcoholic beverages and none of them was taking any medication. All judokas participated in official judo competitions during this year and trained for 16-15 hours per week.

### 2.2. Experimental Design

During the week before the experiment, participants came to the laboratory several times to become fully familiarized with the procedure and tests involved so as to minimize learning effects during the experiment. In a randomized order, subjects participated in two experimental test sessions. The first was a baseline condition in which subjects were taking a normal diet (baseline). The second is a condition of caloric restriction for 7 days; participants reduce their energy intake by 6.7 MJ/day (CR). During each experimental condition, subjects performed the SJFT at the same time of day (08:00 h). All experimental sessions were scheduled during a period with no official competitions.

At the beginning of each session, the body weight, % fat, fat mass, fat-free mass, and body water were recorded using bioelectrical impedance scale to the nearest 0.1 kg (Tanita, Tokyo, Japan) calibrated in accordance with the manufacturer's guidelines by one trained technician. The body mass index (BMI; kg·m^−2^) was then calculated. Following this, duplicate measurements were taken with participants standing and wearing only briefs, as recommended by the guidelines. The average of these two measurements was used for the final analysis. Moreover, the heart rate was monitored using Automatic Blood Pressure (Microlife, W90, Paris). Before the morning test sessions, only one glass (15 to 20 cl) of water was allowed to avoid postprandial thermogenesis effects. Throughout the experimental period, participants were required to maintain their habitual physical activity and to avoid strenuous physical efforts 24 h before each test session.

### 2.3. Assessment of Dietary Intake

Nutritional assessment was carried out every 15 days to assess the energy intake of the subjects (i.e., fat, protein, and carbohydrate). Each subject received recommendations needed to properly complete the food diaries. Thus, they detailed the food and beverages consumed during the 3 days prior to sampling. We asked subjects to maintain their normal diet during the study period (control period). Values for nutrient intakes were obtained from a food diary for a holding period of weight and dietary restriction after 7 days. The plan was to carry out dietary restriction (−6 MJ/day) according to the report from Giannini Artioli et al. [2]. All participants received a detailed verbal explanation and written instructions. The weight loss methods used by athletes in this study appear to be generally used [25]. Values of daily nutritional intake were calculated using dietary assessment software Nutrisoft-Bilnut (ver. 4, Paris, France).

### 2.4. Special Judo Fitness Test (SJFT)

To perform the SJFT, three athletes of similar body mass are needed: a participant (TORI) is evaluated, and 2 other individuals receive throws (UKES). The TORI begins the test in a position between the 2 UKES who are standing 6 m away from one another. On a signal, the TORI runs to one of the UKES and employs a throwing technique called ippon-seoi-nage. The TORI then runs to the other UKE and completes another throw using the same technique. The TORI must complete as many throws as possible within the test time. The SJFT consists of three periods of 15 s (A), 30 s (B), and 30 s (C) separated by 10 s recovery intervals. Performance was determined by the total number of throws completed during each of the SJFTs [21]. In addition, the following index was calculated [23]:

 Index = final HR (bpm) + HR 1 min after the end of the test (bpm)/total number of throws,
 where HR is the heart rate measured immediately after the test and HR 1 is the heart rate measured 1 min after the test.

### 2.5. Blood Samples and Analyses

Blood samples were collected using an indwelling venous catheter before (P1) (after 15 min of rest), immediately (P2), and 60 min after the exercise (P3). Then, plasma tubes were centrifuged at 3000 rpm for 20 min at room temperature and stored at −80°C until the measurement of hormonal and immune parameters [26]. All parameters were measured in triplicate for each sample. Leucocyte counts, lymphocyte, and neutrophil were obtained using an automated blood cell counter (Sysmex Kx-21N, Japan).

GH concentrations were evaluated using the ST AIA-PACK (Tosoh Bioscience). The ST AIA-PACK HGH is a two-site immunoenzymometric assay, which is performed entirely in the AIA-PACK. The intra- and interassay coefficients of variation (CV) were 0.9% and 3.6%, respectively. The limit detection was 0.07 ng/mL.

Testosterone and cortisol concentrations were analysed by ELFA (Enzyme-Linked Fluorescent Assay; VIDAS, BioMerieux, France). The intra- and interassay CV were 18.2% and 5.6% for testosterone and 2.7% and 5.8% for cortisol, respectively. The limit detections were 0.1 ng·mL^−1^ for testosterone and 2 ng·mL^−1^ for cortisol.

IL-6 and TNF-*α* were analyzed by ELISA (Enzyme-Linked Immunoassay) using commercial kits: BE53061, Hamburg, Germany, and AbC223/3, Paris, respectively. The intra- and interassay CV were 3.4% and 5.2% for IL-6 and 6.9% and 7.4% for TNF-*α*, respectively. All assays were carried out as advised by the manufacturer's directions. To eliminate interassay variance, all samples for each subject were assayed in the same assay.

### 2.6. Statistical Analyses

Statistical tests were processed using STATISTICA Software (StatSoft, France). Data were reported as mean ± SD. Once the assumption of normality was confirmed using the Shapiro-Wilk *W*-test, parametric tests were performed. Hormonal and inflammatory mediators data were analyzed using a two-way ANOVA with repeated measures (2 [diet condition] × 3 [points of measurement]). The Bonferroni post hoc test was performed whenever significant effects or a significant interaction was found using ANOVA. Energy intake, anthropometric parameters, performance of SJFT, and heart rate were tested by a paired Student's *t*-test. To assess the data practical significance, effect sizes were calculated as partial eta-squared, *η*_*P*_^2^. Test-retest reliability was assessed by intraclass correlation coefficients (ICCs). The level of statistical significance was set at *P* < 0.05.

## 3. Results

### 3.1. Body Mass and Energy Intake, Performance, and Heart Rate

The statistical analysis showed significant effects of caloric restriction on body weight (*P* < 0.05), fat mass (*P* < 0.05), fat-free mass (*P* < 0.05), and body water (*P* < 0.001) with a significant decrease during CR in comparison to baseline. Likewise, macronutrient intake showed significant decrease during CR in comparison with baseline (*P* < 0.05) statistical differences (Table 1). Likewise, a significant decrease in performance (i.e., total throws) (*P* < 0.05) was observed. The SJFT showed a high reliability between test-retest sessions (ICC higher than 0.87). However, heart rate (i.e., HR immediately (*P* < 0.05) and 1 min after test (*P* < 0.05)) and SJFT index (*P* < 0.05) were significantly higher during CR in comparison to baseline (Table 2).

### 3.2. Hormone Concentrations

**Figure 1:**
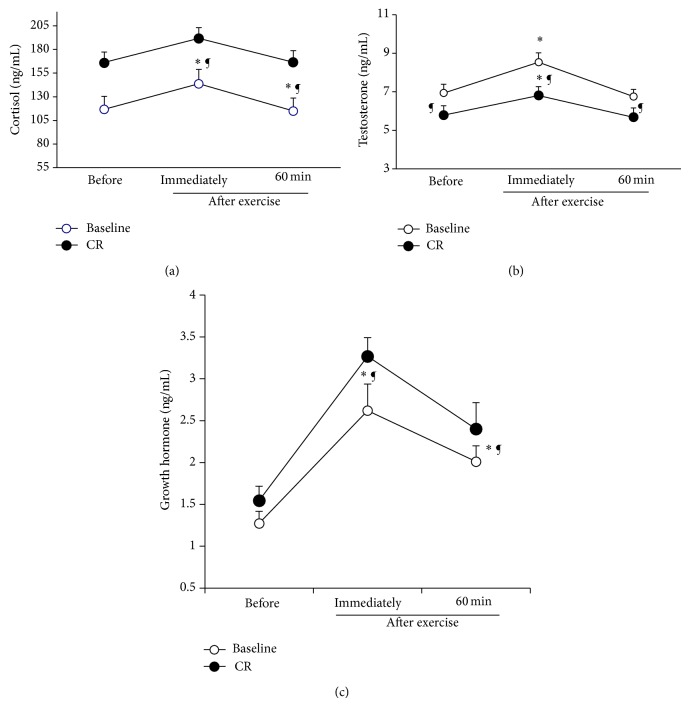
Growth hormone (GH) (a), testosterone (b), and cortisol (c) concentrations (ng·mL^−1^) measured before the exercise (P1) and immediately (P2) and 60 min after (P3) the exercise during baseline and caloric restriction (CR) conditions. All values are expressed as mean (±SD). ∗ indicates significant difference with (P1) during the same condition; ¶ indicates significant difference in comparison with baseline (*P* < 0.05).

Exercise was associated with an increase in testosterone (*F*_(1,10)_ = 5.56; *η*_*P*_^2^ = 0.54; *P* < 0.05), GH (*F*_(1,10)_ = 7.33; *η*_*P*_^2^ = 0.67; *P* < 0.05), and cortisol (*F*_(1,10)_ = 5.20; *η*_*P*_^2^ = 0.59; *P* < 0.05) immediately after the SJFT. Likewise, a significant diet condition effect in testosterone (*F*_(2,20)_ = 63.35; *η*_*P*_^2^ = 0.77; *P* < 0.05), GH (*F*_(2,20)_ = 22.72; *η*_*P*_^2^ = 0.65; *P* < 0.05), and cortisol concentrations (*F*_(2,20)_ = 21.86; *η*_*P*_^2^ = 0.77; *P* < 0.05) was observed (Figure 1). However, there was no significant interaction (diet condition × points of measurement) for testosterone and cortisol concentrations (*P* > 0.05). Moreover, concentrations of testosterone, GH, and cortisol are significantly higher at P2 in comparison with P1 and P3 during baseline and CR (*P* < 0.01). CR leads to a significant decrease in concentrations of testosterone and significant increase in cortisol and GH concentrations at P1, P2, and P3 (*P* < 0.05).

### 3.3. Inflammatory Mediators

**Figure 2:**
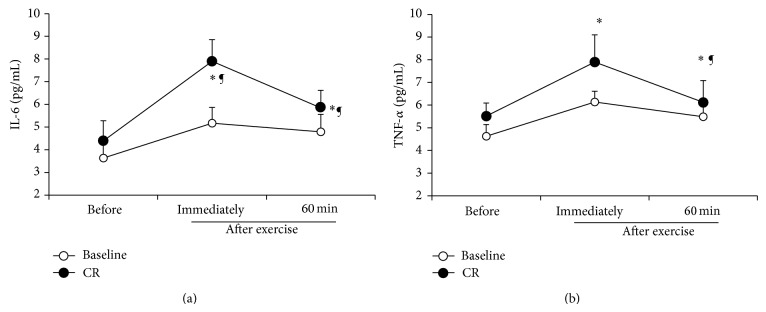
Plasma concentrations (pg/mL) of interleukin-6 (IL-6) (a) and TNF-*α* (b) measured before (P1) and immediately (P2) and 60 min after (P3) the exercise during baseline and caloric restriction (CR) conditions. All values are expressed as mean (±SD). ∗ indicates significant difference with (P1) during the same condition; ¶ indicates significant difference in comparison with baseline (*P* < 0.05).

The effects of CR on inflammatory mediators during the exercise are summarized in Figure 2. Both CR and baseline conditions were associated with increases in IL-6 (*F*_(1,10)_=120.3; *η*_*P*_^2^ = 0.81; *P* < 0.001) and TNF-*α* (*F*_(1,10)_ = 13.53; *η*_*P*_^2^ = 0.67; *P* < 0.01) during P2. A significant diet condition effect on the circulating IL-6 (*F*_(2,20)_=42.38; *η*_*P*_^2^=0.74; *P* < 0.001) and TNF-*α* (*F*_(2,20)_ = 32.04; *η*_*P*_^2^ = 0.69; *P* < 0.001) was observed. However, the interaction diet condition × points of measurement for plasma concentrations of IL-6 and TNF-*α* was not significant (Figure 2). Indeed, the increase in plasma concentrations of IL-6 and TNF-*α* is significantly higher at P2 in comparison with P1 and P3 during CR in comparison with baseline (*P* < 0.05). Leukocytes (*F*_(2,16)_ = 27.53; *η*_*P*_^2^ = 0.65; *P* < 0.001) and lymphocytes (*F*_(2,16)_ = 32.43; *η*_*P*_^2^ = 0.73; *P* < 0.001) during the CR were significantly lower in comparison with baseline whereas neutrophils (*F*_(2,16)_ = 34.16; *η*_*P*_^2^ = 0.71; *P* < 0.001) were significantly higher during the baseline than CR conditions (Table 3). Lymphocytes (*F*_(1,8)_ = 8.6; *η*_*P*_^2^ = 0.67; *P* < 0.01) and neutrophils (*F*_(1,8)_ = 9.7; *η*_*P*_^2^ = 0.66; *P* < 0.01) increased significantly at P2 compared to P1 and remained significantly higher compared to their baseline value. However, leucocytes (*F*_(1,8)_ = 7.77; *η*_*P*_^2^ = 0.63; *P* < 0.01) were significantly lower during CR than baseline at P2.

## 4. Discussion

The results of this study showed that 7 days of CR leads to alterations in immunological responses as well as steroid hormone during a high-intensity specific exercise in judo athletes. Indeed, IL-6, TNF-*α*, GH, testosterone, and cortisol were higher after exercise and remained elevated 60 min after exercise during CR compared to the baseline. Likewise, leukocyte, lymphocyte, and neutrophil counts had a significant variation through exercise during the CR and the baseline conditions.

Food records, as used in this study, are considered the standard for dietary assessment and provide a quantitative account of an individual's diet during a specific period. Therefore, it is important to view the reported data of energy intake for these athletes.

In this study, the caloric intake during the CR period leads to a significant decrease in body weight (4.2 ± 0.5%), which was in accordance with other athletes in combat sports [10, 27]. The loss of body weight represented an average of 3.1 kg in absolute (Table 1). The deficit in energy intake represented about ~6 MJ/day. Overall, our data show that most athletes usually lose weight rapidly before competitions. The average magnitude of weight reductions was around 5% of body weight (i.e., 2–5 times per year). In this context, Artioli et al. [28] observed a ~4% reduction in body weight after a 5-day weight loss period when compared to control values. Likewise, Mendes et al. [29] showed a ~5% reduction in body weight after a 5-day weight loss period. The most frequent methods used by judo players were increased exercise, restricted food ingestion, training in heated rooms, gradual dieting, and fluid restriction [2]. In accordance with several reports our results showed that rapid weight loss affects negatively performance in judo athletes [4, 10].

One can, then, put forward the hypothesis that the main part of the body weight loss observed during the dietary restriction is due to a body water loss. As a result, only 2% of fluid loss induces an increase in the heart rate and lowers the stroke volume, which impairs cardiac output [30].

Our results showed an increase in plasma concentrations of cortisol, testosterone, and GH immediately after the exercise during the two experimental conditions (i.e., baseline and CR). It has, also, been shown that weight reduction may lead to alterations in lipid profile, as it is the case in our study, which may be the consequence of the increased hormonal responses to the exercise. Our data are in agreement with previous studies that showed an increase in testosterone and cortisol immediately after exercise [4, 31]. However, Opstad [32] and Axelsson et al. [33] found no change in cortisol concentrations. The conflicting findings may be attributed to the differences in the intensity and duration of exercise.

More to the point, restrained eating may be a stressor which induced hormonal changes (i.e., decrease of testosterone and increase of cortisol). These data are in accordance with the results of Anderson et al. [34]. The low carbohydrate intake may affect the cortisol levels [35]. Therefore, higher concentrations of cortisol may be a main factor in the susceptibility to infections and injuries because of its immunosuppressive role [36]. By contrast, measurements of testosterone showed decreases with diet and fluid restriction similar to those reported in several studies [37, 38]. Strauss et al. [38] reported significant relationships between testosterone values, weight loss, body fat %, and body fat loss. It may be possible that the dehydration-induced weight loss and caloric restriction experienced by the judo athletes in the present study combined with the high-intensity exercise contributed to the reduced testosterone concentrations.

Moreover, Loucks and Thuma [39] showed increase in GH, IGFBP1, and cortisol concentrations and a decrease in the plasma concentration of IGF-1. Nindl et al. [31] observed that brief periods of intense physical activity superimposed on energy and sleep restriction induced amplifications in GH and LH secretions. However, due to methodological differences such as exercise type (e.g., intensity and duration) or sleep loss protocol, the present study's results cannot be compared with those of previous investigations.

To our knowledge, this study is the first to report that high-intensity exercise induced changes on proinflammatory cytokines (i.e., IL-6 and TNF-*α*) during 7 days of CR. Judo is characterised by high-intensity, short-duration exercise and it requires the athlete to use a combination of aerobic and anaerobic capacities.

Our results showed that plasma concentrations of IL-6 and TNF-*α* increased significantly during the exercise and remained elevated during the recovery period (i.e., 60 min after the exercise) after CR. This report confirms cytokines as effective markers of normal recovery after intense exercise.

Consistently, previous reports showed that high-intensity exercise was associated with increase of proinflammatory cytokines, for example, IL-6 and TNF-*α* [40–42], which have been proposed as mediators of pathologies in humans and have been shown to be associated with an unfavourable metabolic profile with implications for inflammation and cardiovascular disease risk [12]. Furthermore, exercise induces a general stress response involving the activation of the immune systems and may induce immunosuppression in the recovery period and continues to increase after exercise [12, 41]. Additionally, the concentrations of TNF-*α* have been shown to increase by 2-3-fold after exercise [43]. The major source for the exercise-related IL-6 increase is the skeletal muscle [44].

Another explanation suggested that glycogen depletion, a major stimulus for IL-6 release [42], caused by muscle damage was a further stimulus for the elevation of IL-6 during the recovery phase during CR condition. Furthermore, elevated plasma concentrations of IL-6 have been associated with the decrease of athletic performance and, therefore, increased levels of fatigue [45]. Regarding the effect of CR on immune parameters, our results showed a significant increase in plasma concentrations of IL-6 and TNF-*α* following the 7 days of CR. These results are in agreement with previous reports that showed an alteration of the various components of the immune system. Imai et al. [16] showed that weight loss decreased T-cell proliferation and production of interleukin-2 and interferon-g, whose cytokines induce T-cell activation and proliferation in wrestling athletes. In turn, fluid deficits associated with intensive exercise, particularly in hot environments, might be at least partly responsible for the alteration of immune function. Therefore, weight loss in athletes might impair T-cell activation. In highly trained athletes, URTIs are common reported illness [14]. Consequently, performing high-intensity exercise or periods of strenuous exercise training is associated with impairment of immune function and a high risk of URTI in elite judo athletes. In this study, the increase of proinflammatory cytokines might be related to susceptibility to infection.

### 4.1. Study Limitation

This study has some limitations. There was a smaller sample size of the subjects that limited our power to perform analyses. In addition, no control subjects without weight loss and without psychological stress related to an upcoming competition participated in this study. Additional studies must use proper number of weight loss and control subjects who are expending energy similar to that used for judo (e.g., wrestling) but who are not participating in the competition, who are not pursuing weight loss such as open-category class athletes (e.g., 100 kg class in judo), and who perform either fluid restriction program or dietary energy restriction program. In addition, we did not measure direct dehydration status such as urine specific gravity. Future study needs to examine the influences of weight loss on immune parameters and somatotropic axis (i.e., IGF-1) in parallel with hydration status.

## 5. Conclusion

In conclusion, we conclude that 7 days of weight loss programs undertaken in preparation for competition might degrade immune functions and conduct to the appearance of infectious diseases such as URTI symptoms in male judo athletes by the increase of proinflammatory cytokines (i.e., IL-6; TNF-*α*). Despite conducting weight loss activities to achieve an advantage in competition, the consequent weight loss might impair immune functions as well as endocrine functions, thereby depressing the overall physical condition. Therefore, it is necessary for athletes to develop safer and more effective weight loss programs. As a result, athletes, coaches, and medical staff should be sensitive to periods of increased risk of disease (such as weeks of intensive training, the tapering period before the competition, and during the competition) and pay particular attention to recovery, the nutritional strategies, and optimal fluid intake and should monitor athletes' body weight, hydrated status, and immunocompetence to prevent their physical condition from becoming worse.

## Figures and Tables

**Table 1:** Mean (±SD) values for daily nutrient consumption and anthropometrics parameters during baseline and CR conditions.

	Baseline	CR
Body mass (kg)	75.9 ± 3.1	72.73 ± 3.1^*^
BMI (Kg·m^2^)	25.8 ± 1.1	24.7 ± 1.01^*^
Body fat (kg)	12.09 ± 1.09	10.84 ± 1.5^*^
FFM (Kg)	63.8 ± 1.1	61.8 ± 1.5^*^
Body water (Kg)	47.72 ± 1.9	41.8 ± 1.8^*^
Energy intake (Kcal/day)	3475 ± 247.6	2192 ± 235.6^*^
Protein (g/day)	129.2 ± 17.2	87.4 ± 9.2^*^
Fat (g/day)	117.6 ± 12.6	89.5 ± 7.7^*^
Carbohydrates (g/day)	355.7 ± 32.7	234.4 ± 46.7^*^

^*^Significant difference: *P* < 0.05 versus baseline.

FFM: free fat mass; BMI: body mass index.

**Table 2:** Mean (±SD) results of SJFT (i.e., performance and heart rate (HR)) in judo athletes during baseline and CR conditions.

	Baseline	CR
Total throws (1 + 2 + 3)	31 ± 2.65	26.27 ± 2.9^*^
HR immediately after exercise	182.3 ± 5.3	188 ± 8.4^*^
HR after 1 min	154 ± 2.8	161 ± 3.2^*^
SJFT index	10.8 ± 3	13.28 ± 4^*^

^*^Significant difference: *P* < 0.05 versus baseline.

**Table 3:** Changes of hematological measurements measured before the exercise (P1) and immediately (P2) and 60 min after the exercise (P3) during baseline and CR.

	Baseline			CR		
	P1	P2	P3	P1	P2	P3
Leucocyte (10^3^/mm^3^)	7.4 ± 0.39	5.4 ± 0.87^*^	5.9 ± 0.166	6.5 ± 0.53^¶^	4.7 ± 0.47^∗¶^	4.5 ± 0.73^¶^
Lymphocyte (%)	39.2 ± 5.2	46.61 ± 3.4^*^	40.16 ± 2.4	36.2 ± 2.9^¶^	38.3 ± 4.7^∗¶^	34.2 ± 5.3^¶^
Neutrophil (%)	47.3 ± 4.3	50.8 ± 3.2^*^	53.3 ± 2.41	49.4 ± 2.9^¶^	55.4 ± 4.7^∗¶^	59.9 ± 5.3^¶^

^*^Significant difference: *P* < 0.05 versus P1.

^¶^Significant difference: *P* < 0.05 versus baseline.

## Abbreviations

SJFT: Special Judo Fitness Test
GH: Growth hormone
CR: Caloric restriction
URTIs: Upper respiratory tract infections
NK: Natural killer cells
IL-6: Interleukin-6
TNF-*α*: Tumour necrosis factor-alpha.
